# Starved and Asphyxiated: How Can CD8^+^ T Cells within a Tumor Microenvironment Prevent Tumor Progression

**DOI:** 10.3389/fimmu.2016.00032

**Published:** 2016-02-10

**Authors:** Ying Zhang, Hildegund C. J. Ertl

**Affiliations:** ^1^Gene Therapy and Vaccines Program, University of Pennsylvania School of Medicine, Philadelphia, PA, USA; ^2^The Wistar Institute Vaccine Center, Philadelphia, PA, USA

**Keywords:** lack of glucose, hypoxia, CD8^+^ T cell metabolism, metabolic stress, tumor-infiltrating lymphocytes, tumor microenvironment, fatty acid metabolism, ketone bodies

## Abstract

Although cancer immunotherapy has achieved significant breakthroughs in recent years, its overall efficacy remains limited in the majority of patients. One major barrier is exhaustion of tumor antigen-specific CD8^+^ tumor-infiltrating lymphocytes (TILs), which conventionally has been attributed to persistent stimulation with antigen within the tumor microenvironment (TME). A series of recent studies have highlighted that the TME poses significant metabolic challenges to TILs, which may contribute to their functional exhaustion. Hypoxia increases the expression of coinhibitors on activated CD8^+^ T cells, which in general reduces the T cells’ effector functions. It also impairs the cells’ ability to gain energy through oxidative phosphorylation. Glucose limitation increases the expression of programed cell death protein-1 and reduces functions of activated CD8^+^ T cells. A combination of hypoxia and hypoglycemia, as is common in solid tumors, places CD8^+^ TILs at dual metabolic jeopardy by affecting both major pathways of energy production. Recently, a number of studies addressed the effects of metabolic stress on modulating CD8^+^ T cell metabolism, differentiation, and functions. Here, we discuss recent findings on how different types of metabolic stress within the TME shape the tumor-killing capacity of CD8^+^ T cells. We propose that manipulating the metabolism of TILs to more efficiently utilize nutrients, especially during intermittent periods of hypoxia could maximize their performance, prolong their survival and improve the efficacy of active cancer immunotherapy.

## Introduction

Following antigenic stimulation, differentiation of naive CD8^+^ T cells into effector cells is accompanied by metabolic reprograming to accommodate their increased demand for energy and biomass formation. Resting CD8^+^ T cells primarily gain energy through oxidative phosphorylation (OXPHOS), the mitochondrial pathway of energy production (1). The tricarboxylic acid (TCA) cycle, which is linked to OXPHOS, oxidizes acetyl-CoA. This metabolite can be derived from carbohydrates, amino acids, or FAs. Upon CD8^+^ T cell activation, T cell receptor (TCR) and costimulator CD28 ligation activate the phosphatidylinositol 3-kinase (PI3K)/protein kinase B (Akt)/mammalian target of rapamycin (mTOR) pathways, which in turn increase the activity of hypoxia-inducible factor (HIF)-1α and Myc (2). HIF-1α augments surface expression of Glut1, and thereby allows for increased uptake of glucose. Akt and Myc increase the activity of several glycolytic enzymes, whereas PI3K activation reduces expression of enzymes of the TCA cycle and OXPHOS (3–5). All of these signals drive CD8^+^ T cells to rely increasingly on glycolysis after activation (6). Glycolysis is less efficient but faster in generating ATP and provides building blocks to support cell proliferation and effector functions.

Once T cells are past the acute activation phase, they resume energy production through the TCA cycle and OXPHOS, which is partially supported by fatty acid oxidation (FAO) (7, 8). One facilitator of this metabolic switch is increased expression of PD-1, a coinhibitor that blocks CD28-mediated activation of the mTOR pathway (9) and reduces glycolysis and promotes lipid metabolism (10). PD-1 thereby prevents overwhelming CD8^+^ T cell activities and promotes memory formation (11).

Increased expression of PD-1 also identifies the so-called exhausted CD8^+^ T cells, which evolve during chronic infections due to continuous antigen-driven stimulation (12). Exhausted CD8^+^ T cells show progressive increases in the expression of additional coinhibitors, such as lymphocyte-activation gene 3 (LAG-3), CD160, 2B4, and others, which combined with loss of effector functions eventually lead to cell death (13). Within the tumor microenvironment (TME), CD8^+^ T cells become exhausted (14). It is assumed that lack of an effective antitumor T cell response is at least in part linked to exhaustion. Consequently, blockade of coinhibitor pathways by antibodies such as to anti-PD-1 or anti-PD-L1 has yielded promising results in some cancer patients (15).

The etiology of T cell exhaustion within the TME warrants further discussion. High level of antigen as found during chronic viral infections may not be solely responsible. The amount of antigens within a tumor would not be expected to be overwhelming, especially as tumor cells commonly downregulate MHC class I expression (16), which makes their antigens virtually invisible to CD8^+^ T cells. Instead, we propose that CD8^+^ T cell exhaustion may be triggered by metabolic stress within the TME. Cancer cells ferociously consume glucose to fuel energy production through glycolysis, which can lead to hypoglycemia within tumors. In addition, angiogenesis often lags behind expansion of solid tumors, leading to hypoxia in some areas of the TME. T cells that infiltrate solid tumors thus face dual metabolic jeopardy; lack of glucose prohibits energy production through glycolysis, whereas lack of O_2_ prevents energy production through OXPHOS. How tumor-specific CD8^+^ tumor-infiltrating lymphocytes (TILs) cope with these challenges and how metabolic reprograming of CD8^+^ TILs will affect their antitumor performance require further investigations, in order to improve the efficacy of cancer immunotherapy.

## Effects of Hypoxia on CD8^+^ TIL Metabolism and Functions

Solid tumors commonly have areas of hypoxia. This can be caused by lack of perfusion due to structural and functional abnormalities of the tumor microvasculature, general anemia of the patient, or insufficient diffusion due to lack of angiogenesis. The latter affects cells once they are more than 70 μm away from a blood vessel. Studies have shown that up to 50–60% of solid tumors of a variety of different types possess unevenly distributed areas of hypoxia (17).

Tumor-infiltrating CD8^+^ T cells are initially activated under physiological O_2_ tension in peripheral lymphatic tissues. Upon tumor entry, CD8^+^ T cells will be subjected to increasingly severe hypoxia once they leave areas close to blood vessels; this will activate HIF-1α. HIF-1α signaling adjusts the cells’ metabolism to allow for energy production when O_2_ is limiting. HIF-1α enhances glycolysis by CD8^+^ T cells mainly by promoting the activity of lactate dehydrogenase A (LDHa), while inhibits OXHPOS by increasing expression of pyruvate dehydrogenase kinase 1 (PDK1), which prevents the oxidation of pyruvate to acetyl-CoA (18, 19). CD8^+^ TILs under hypoxia therefore must increase glucose consumption to fuel glycolysis.

A series of *in vitro* and *in vivo* studies in the past two decades show that hypoxia dampens lymphocyte activation, diminishes their proliferation, and reduces the ability of activated T cells to produce cytokines or lytic enzymes (20–24). T cell activation causes release of Ca^2+^ from intracellular stores followed by sustained Ca^2+^ influx, which is inhibited by increased HIF-1α activity (25). Whole body hypoxia dampens inflammation and T cell functions in mice and humans (26, 27). These data show that hypoxia is immunosuppressive and metabolic reprograming due to increased activity of HIF-1α may contribute to reductions of immune responses. This could be caused by reduced ATP production due to impaired OXPHOS under hypoxia. Additionally, hypoxia is known to increase accumulation of reactive oxygen species (ROS), which may induce apoptosis of activated T cells (28, 29)*. Vice versa*, activated CD8^+^ T cells with a partial deficiency in HIF-1α show enhanced production of cytokines (30, 31). One study has shown that under hypoxia or with increased HIF-1α activity T cells increase expression of coinhibitors, including CTLA-4, CD244, and LAG-3, and decrease levels of T-bet (32), a key transcription factor that controls many of the T cells’ functions, again indicating that hypoxia is immunosuppressive.

Conversely, two recent reports show that hypoxia and increases in HIF-1α activity promote effector T cell functions, especially production of the lytic enzymes granzyme B and perforin (32, 33). There is a caveat with these studies; both used a protocol in which after an initial 48 h of activation, CD8^+^ T cells were rested for several days in IL-2-supplemented medium before being subjected to hypoxia. Unlike highly activated CD8^+^ T cells, resting CD8^+^ T cells rely more on FAO and OXPHOS for energy production. This metabolic reprograming and their decreased energy demand may allow CD8^+^ T cells to improve some functions upon hypoxia. In real life CD8^+^ T cells induced by a cancer vaccine or tumor antigens (TAs) that leaked into lymphatic tissues are unlikely to rest before they infiltrate a tumor, where they may receive additional activation signals. Results obtained with resting cells are thus not pertinent to TILs exposed to hypoxia. The same papers showed that genetic depletion of HIF-1α reduces CD8^+^ T cell functions while its constitutive overexpression through functional depletion of the Von Hippel–Lindau (VHL) factor improves functions. During the initial phase of T cell activation, HIF-1α is essential to allow T cells to use glycolysis. The effects of its complete absence or increased expression during this critical phase of differentiation may differ from changes in HIF-1α activity during later phases of activation. These studies thus give limited insights into the effect of hypoxia or HIF-1α on CD8^+^ TILs, which encounter limited O_2_ after activation in the periphery once they penetrate into the tumor.

Hypoxia and increased HIF-1α activity in tumor tissues in general correlate with poor prognosis of cancer patients (34, 35). Hypoxia not only affects protective immune responses but also promotes tumorigenesis by enhancing proliferation of cancer cells and increasing their PD-L1 surface expression (36). The latter in turn may further dampen functions and survival of PD-1^+^ TILs. Hypoxia may also increase the suppressive activity of tumor-infiltrating myeloid suppressor cells and tumor-associated macrophages, which will lead to further impairments of CD8^+^ TIL functions (37, 38). Overall, all of these studies strongly suggest that lack of O_2_ negatively affects metabolism and functions of CD8^+^ TILs.

## Effects of Hypoglycemia on Metabolism and Functions of TILs

Glucose is crucial during the initial stages of CD8^+^ T cell activation. Naive CD8^+^ T cells can differentiate into effectors in absence of glucose but then become functionally impaired (39). Lack of glucose also dampens effector functions of fully activated CD8^+^ T cells both *in vitro* and *in vivo* (39–44).

Attracted by chemokines, activated CD8^+^ T cells regardless of their antigen specificity infiltrate solid tumors. Here, they encounter an environment where key nutrients such as glucose may be limiting due to its consumption by tumor cells (45). Although activated CD8^+^ T cells express increased levels of the glucose transporter Glut1, *in vitro* studies show that their effort to take up glucose is thwarted by tumor cells, which are simply more effective at consuming this key nutrient (39). CD8^+^ T cell glycolysis within TME may further be reduced by accumulating concentrations of tumor cell-derived lactate, which prevents the monocarboxylate transporter-1-mediated, gradient-dependent export of lactate from CD8^+^ T cells. Increasing concentration of lactate within CD8^+^ T cells in turn causes a fall in pH, which inhibits the activity of phosphofructokinase, a key enzyme of glycolysis (46). In addition, glucose deprivation increases coinhibitor PD-1 expression on activated CD8^+^ T cells (47), which can further reduce glycolysis but enhance FA metabolism. Blockade of PD-1 has been shown to lessen the CD8^+^ TILs’ metabolic stress by augmenting their glycolytic capacity through increased mTOR signaling (39).

It has been reported that FAO can maintain the survival of cancer cells when glucose is not available (48). T cells may also be able to cope with lack of glucose by enhancing other metabolic pathways. Sudden deprivation of glucose can lead to drops in ATP with enhanced AMP in activated CD8^+^ T cells. The increased AMP:ATP ratio activates the energy sensor AMP-activated protein kinase (AMPK). AMPK is a key regulator that reduces the T cells’ energy expenditure by blocking production of cytokines (49). Furthermore, AMPK maintains T cell viability by decreasing glycolysis and anabolic processes through inhibition of the mTOR pathway, while enhancing OXPHOS fueled by FAs and glutamine (50, 51). In agreement, the studies showed that knockout of AMPK increases apoptosis of T cells activated with limited access to glucose (49).

To what degree CD8^+^ TILs’ functions are impaired by lack of glucose within the TME may depend on the T cells’ differentiation status, or, in other words, on their metabolic programing prior to enter the tumors. Recently activated CD8^+^ effector T cells conditioned to use glycolysis are likely most susceptible to sudden loss of exogenous glucose (52, 53), as compensatory endogenous production of glucose through gluconeogenesis or glycogen degradation are not sustainable (54). By contrast, CD8^+^ T cells programed to use other nutrients may cope better with restricted glucose access (55). This in turn invites the testing of metabolic drugs that reprogram T cell metabolism as adjuvant treatments for active cancer immunotherapy or adoptively transferred TA-specific T cells.

## Effect of Hypoxia Combined with Hypoglycemia on CD8^+^ TIL Functions and Metabolism

Hypoxia inhibits OXPHOS but allows cells to gain energy through glycolysis. On the other hand, hypoglycemia reduces glycolysis but cells can switch to OXPHOS by burning other nutrients. The problem is that many tumors have low levels of glucose combined with areas of hypoxia, which foils both pathways of energy production.

Malignant cancer cells increase lipogenesis, lipolysis, FA secretion, and recruit adipose progenitors to the TME (56–58). In addition, dying tumor cells may release FAs. FAs provide ample energy through peroxisomal or mitochondrial FAO, which may be used by CD8^+^ TILs. FAO is preferred by some effector T cells such as those participating in graft versus host disease (59), suggesting that FAO may be preferred by T cells that encounter large quantities of antigens (60). Energy production through FAs requires more O_2_ than energy production through glucose to generate equivalent amounts of ATP. OXPHOS fueled by glucose yields 36 molecules of ATP and consumes 6 molecules of O_2_. By contrast, OXPHOS fueled by palmitate, a 16-carbon FA, results in a net yield of 129 ATP and requires 31 molecules of O_2_. FAs thus require 1.44 times more O_2_ than glucose to provide the same amount of energy, which makes it an inefficient fuel within an O_2_-deprived TME.

Ketone bodies, i.e., acetoacetate, acetone, and β-hydroxybutyrate, are produced during FA catabolism when the amount of acetyl-CoA produced by FAO overwhelms the processing capacity of the TCA cycle. When acetyl-CoA declines, ketone bodies can be converted back to acetyl-CoA providing a ready source of energy that requires less O_2_ than catabolism of FAs (61). Previous studies showed that under conditions of hypoxia and hypoglycemia, cells of the nervous system maintain their energy balance through the use of ketone bodies (62). We propose that CD8^+^ TILs may do the same. T cells may take up ketone bodies from the surrounding or they could synthesize them directly (61, 63, 64).

CD8^+^ TILs are not stationary; they migrate throughout the TME (65) and we assume that their environment changes accordingly. When T cells are close to vessels and O_2_ is readily available, FAs may fuel the TCA cycle and excess FA-derived acetyl-CoA can be converted into ketone bodies. When T cells penetrate deeply into the tumor and O_2_ becomes scarce, T cells may burn ketone bodies to sustain their energy requirement. As has been shown during heart ischemia, lack of O_2_ results in increases in AMPK, which decreases the activity of acetyl-CoA carboxylase (ACC) that converts acetyl-CoA to malonyl-CoA. Once cells have access to O_2_, such upon reperfusion of an ischemic heart or migration of TILs to areas close to vasculature, lack of malonyl-CoA will cause a surge in FAO (66). Lipid metabolism has been shown to correlate with long-term survival in many different cell types (67, 68). It has been suggested that PD-1 ligation, which prevents terminal differentiation of effector CD8^+^ T cells (69), promotes survival by enhancing the cells’ FA metabolism (10). Ketone body metabolism also promotes metabolic fitness and longevity of cells by regulating histone deacetylase (HDAC) activities (61). Catabolism of these carbon sources may allow T cells to survive under hypoglycemia and intermittent hypoxia.

## Exploring Metabolic Manipulations to Improve T Cell-Mediated Immunotherapy of Cancer

Adoptive transfer of *ex vivo* expanded TILs has achieved some successes in treatment of melanomas (70). Alternatively, T cells from peripheral blood can be modified to express chimeric antigen receptors (CARs) that recognize cell surface expressed TAs independent of major histocompatibility antigens (71). Transfer of such CAR-T cells has been remarkably successful in treatment of acute lymphatic leukemia or B cell lymphoma but in general yielded disappointing results in patients with solid tumors (72–76). This has been blamed on the immunosuppressive nature of the TME (77, 78). Treatments that reduce numbers or functions of regulatory T cells, myeloid suppressor cells, tumor-associated macrophages, or that block immune checkpoints have improved the efficacy of adoptive cell transfer for cancer therapy (79, 80).

As already mentioned, the metabolic profile of CD8^+^ T cells prior to their tumor infiltration has significant impacts on their longevity and performance within TME. In addition, the metabolic profiles of the tumor cells will influence what nutrients are available to T cells. Some studies show that drugs, which inhibit glycolysis by tumor cells such as Glut1 inhibitors, cause tumor regression and increase glucose supply within the TME (81, 82). Its use prior to cell transfer could increase the efficacy of cell immunotherapy. Others have shown that reducing glucose consumption by tumor cells through blockade of PD-L1 signaling allows for increased glycolytic energy production by CD8^+^ TILs, which is accompanied by improvements of their functions (39). A recent study shows that glucose limitation leads to reduction of the glycolysis metabolite phosphoenoylpruvate (PEP), which is essential for Ca^2+^-NFAT signaling in CD8^+^ TILs. Overexpressing phosphoenolpyruvate carboxykinase (PCK) 1, which converts the TCA cycle intermediate oxaloacetate to PEP, was shown to improve NFAT signaling and function of TILs (44).

However, glycolysis may accelerate terminal differentiation of CD8^+^ T cells, and thereby shorten T cell survival (83); thereby, limiting glycolysis by CD8^+^ TILs may yield better therapeutic effects. One study showed that inhibition of glycolysis by 2-deoxyglucose during *in vitro* expansion of TA-specific CD8^+^ T cells increases their efficacy in a mouse melanoma model (52). A similar effect was seen when TILs were treated prior to adoptive transfer with an Akt inhibitor, which reduces their use of glycolysis and increases OXPHOS (57). Along the same line, when CD8^+^ T cells were cultured *in vitro* with IL-7 or IL-15, which drives their differentiation toward memory, their antitumor efficacies *in vivo* significantly improved (84–86). Further studies showed that adoptive transfer of central memory CD8^+^ T cells confers better eradication of solid tumor masses than transfer of equal numbers of effector CD8^+^ T cells (87, 88), which led the authors to conclude that the T cell differentiation status, which dictates potential for proliferation, is crucial to ensure optimal efficacy of adoptive T cell transfer. We think that the metabolic reprograming toward preferential use of FAO and OXPHOS as naturally occurs during differentiation from effector to memory cells, may allow for the superior performance of the transferred T cells within TME.

## Conclusion

Immunotherapy of cancer is still in its infancy. T cells are able to stop an infection by rapidly killing millions of infected cells but they clearly need additional help to eliminate tumors. In recent years, exciting new studies have started to illuminate the metabolism of T cells under different conditions and its impact on T differentiation and functions. A better understanding of the metabolic programs utilized by CD8^+^ TILs and how they affect the TILs’ antitumor performance is crucial to find new therapeutic targets for cancer immunotherapy. Metabolic manipulations that could prepare TA-specific T cells, which are either induced by cancer vaccines or expanded *ex vivo* in form of TILs or CAR-T cells, to optimally cope with the metabolic constrains within the TME may significantly improve the overall antitumor efficacy. Undoubtedly, the type of cancer and peculiarities of its TME will dictate the most suited metabolic treatment.

## Author Contributions

YZ: drafted and edited the manuscript; HE: edited and approved the final version of the manuscript.

## Conflict of Interest Statement

The authors declare that the research was conducted in the absence of any commercial or financial relationships that could be construed as a potential conflict of interest.
